# Time-Domain Analysis of Rectangular Pulse Response in Capacitive Impedance Sensing Using Capacitively Coupled Contactless Electrodes

**DOI:** 10.3390/s26133999

**Published:** 2026-06-24

**Authors:** Damian Wanta, Waldemar T. Smolik, Mikhail Ivanenko, Jacek Kryszyn, Oliwia Makowiecka, Grzegorz Domański, Przemysław Wróblewski, Mateusz Midura, Mateusz Orzechowski

**Affiliations:** 1 Faculty of Electronics and Information Technology, Warsaw University of Technology, Nowowiejska 15/19, 00-665 Warsaw, Poland; waldemar.smolik@pw.edu.pl (W.T.S.); mikhail.ivanenko.dokt@pw.edu.pl (M.I.); jacek.kryszyn@pw.edu.pl (J.K.); grzegorz.domanski@pw.edu.pl (G.D.); przemyslaw.wroblewski@pw.edu.pl (P.W.); mateusz.midura@pw.edu.pl (M.M.); mateusz.orzechowski@pw.edu.pl (M.O.); 2 PIT-RADWAR S.A., Poligonowa 30, 04-051 Warsaw, Poland; o.makowiecka@pitradwar.com

**Keywords:** impedance measurement, capacitively coupled electrodes, machine learning, two-electrode configuration

## Abstract

Impulse-based impedance sensing with capacitively coupled electrodes is introduced as a fast, non-contact, and simplified complementary method to conventional capacitive impedance measurements. Unlike frequency-domain methods, the proposed approach derives effective resistive and capacitive properties of a sample from the transient response to a single rectangular pulse. The equivalent circuit model comprises three elements: sample resistance, sample capacitance, and electrode coupling capacitance. From this model, analytical expressions of the transient response were derived, enabling accurate simulation of measured signals and providing the basis for both phantom verification and machine learning training. Importantly, the coupling capacitance, typically considered a limitation in contactless methods, is estimated alongside the sample parameters, providing insight into electrode–object coupling conditions. A machine-learning model trained on simulated circuit responses, including noise and temporal variability, is employed as a low-latency estimator for extracting parameters from measured transient signals. Experimental validation was carried out using a configurable lumped-element equivalent circuit and NaCl solutions of controlled conductivity, cross-verified with conductometric measurements and numerical probe simulations. Across a tested conductivity range, the method achieved estimation errors of 2–8%. The proposed approach is intended as a low-latency measurement strategy for simplified capacitively coupled impedance sensing, with potential relevance to future capacitively coupled electrical impedance tomography systems, where rapid acquisition of boundary measurements is prioritized over full frequency-resolved impedance spectroscopy.

## 1. Introduction

Impedance measurement is a fundamental technique for characterizing the electrical properties of materials, fluids, and biological tissues. It provides access to both resistive and capacitive components of a system and is a key tool in electrochemistry, materials science, biomedical monitoring, and industrial sensing applications [1,2]. In biomedical engineering, impedance spectroscopy has been applied to assess tissue health, detect physiological changes, and evaluate hydration levels [3,4,5]. In electrochemistry, impedance analysis supports the characterization of batteries, fuel cells, and sensors [6]. In fluid monitoring, it enables non-invasive estimation of ion concentration and conductivity [7]. These diverse applications create demand for impedance measurement methods that offer a well-defined compromise between accuracy, repeatability, dynamic range, noise immunity, and compatibility with the electrical and chemical properties of the tested object.

Frequency-domain techniques, most notably electrochemical impedance spectroscopy (EIS), remain the reference approach when a system must be characterized over a broad frequency range and when separate access to resistive, capacitive, charge-transfer, or diffusion-related processes is required [8,9,10]. Their practical performance is typically assessed in terms of accuracy, phase resolution, repeatability, dynamic range, and immunity to drift and noise. The acquisition time of an EIS scan is largely determined by the lowest frequency included in the sweep and by the required signal averaging [11]. In addition, although compact integrated solutions are commercially available, a complete EIS measurement chain still relies on sinusoidal excitation, synchronized voltage/current acquisition, and phase-sensitive processing across multiple frequency points. In applications where only a small set of lumped parameters is needed, these requirements may be unnecessarily demanding. Other instruments, such as LCR meters and bridge-based analyzers, can simplify the implementation, but they still operate at discrete frequencies rather than from a single broadband transient [12].

Although full bioimpedance spectroscopy provides detailed frequency-dependent information about biological tissues and electrochemical interfaces, such complete spectral characterization is not always required in imaging-oriented applications. In capacitively coupled electrical impedance tomography (CCEIT), the primary objective is to acquire many boundary measurements rapidly for image reconstruction [13]. Therefore, simplified time-domain measurement strategies may be useful when low latency is more important than full frequency-resolved impedance characterization.

In galvanic-contact impedance measurements, direct metal–medium contact may introduce electrode polarization, interfacial impedance, electrochemical reactions, corrosion, sample contamination, and sensitivity to electrode material and surface condition [14,15,16]. To mitigate these effects, capacitively coupled methods have been proposed [17,18]. For example, capacitively coupled contactless conductivity detection (C4D) is widely used in capillary electrophoresis and microfluidics [19,20]. In C4D, electrodes are separated from the medium by a dielectric barrier, and conductivity is inferred through capacitive coupling [21]. While C4D avoids direct contact, the unknown and variable coupling capacitance often compromises accuracy and is rarely modeled explicitly in equivalent circuits [13,22]. This coupling is often treated as a nuisance parameter and excluded from equivalent circuit models [20]. As a result, careful calibration is typically required, and measurement repeatability remains limited [23,24].

Aside from classical frequency-domain techniques, time-domain methods have been explored as an alternative approach to impedance characterization [25]. Instead of sweeping multiple frequencies, they analyze transient responses to step or pulse excitation and can, in principle, yield impedance information more rapidly [3,26,27,28,29]. These approaches should also be distinguished from time-domain reflectometry (TDR), where impedance information is typically inferred from the propagation and reflection of fast electrical waves along a transmission line [30]. In contrast, lumped-element transient analysis describes the measured waveform using an equivalent circuit, without explicitly analyzing propagation delays or reflections from impedance discontinuities. Although previous time-domain and pulse-based methods demonstrate the usefulness of transient excitation for rapid impedance-related sensing, the electrode–sample interface is typically galvanic, or the coupling capacitance is not treated as an explicit unknown parameter estimated together with the sample resistance and capacitance. This motivates the development of time-domain measurement models in which the coupling capacitance is treated as an explicit part of the contactless sensing configuration.

The electrode configuration strongly influences impedance measurements [31]. In biomedical and electrochemical contexts, four-electrode setups are commonly used to minimize the influence of electrode contact impedance [32,33]. Two-electrode arrangements are simpler and more compact but suffer from larger uncertainty due to electrode polarization and interface effects [34]. In capacitively coupled systems, two-electrode configurations are more common, as the dielectric barrier already decouples the electrodes from the sample [35]. The interaction between sample impedance and electrode coupling capacitance remains insufficiently characterized in the impedance measurement literature [36], although this topic has been considered, for example, in ECG-related studies [37].

Recent years have seen a growing interest in applying machine learning to impedance measurements and related sensing techniques [6,38,39]. Neural networks have been used to fit nonlinear equivalent circuit models, classify material properties, or denoise measurement signals. Such approaches improve robustness against noise, timing misalignments, and nonidealities in measurement setups. While ML-based analysis has been applied to classical EIS or biosignals, its use in time-domain, capacitively coupled measurements has not yet been sufficiently explored.

To summarize, current impedance measurement methods face several limitations. EIS and related alternating current (AC) techniques are accurate but slow, contact-dependent, and calibration-intensive. C4D and other capacitive approaches avoid galvanic contact, but the coupling capacitance is often not treated as an explicit estimated parameter, which may reduce accuracy and reproducibility. Time-domain methods promise faster measurements but have rarely been applied in contactless configurations [40]. Moreover, no method to date combines impulse excitation, explicit modeling of coupling capacitance, and machine learning-based parameter estimation in a two-electrode capacitive setup.

In this paper, we introduce an impulse-based capacitive impedance measurement method employing capacitively coupled electrodes. An analytical equivalent circuit model is derived, explicitly including the coupling capacitance alongside the sample resistance and capacitance. This model enables accurate simulation of measured signals and forms the basis for parameter estimation. Since the coupling capacitance influences both the transient waveform and the identifiable parameter range, it is treated as an estimated parameter rather than as an unmonitored nuisance factor.

The present implementation is evaluated using a simplified lumped equivalent-circuit model that captures the effective sample resistance, sample capacitance, and electrode coupling capacitance. This model is suitable for validating the proposed single-pulse measurement concept under controlled conditions, but realistic biological tissues and physiological fluids may exhibit dispersive and non-ideal impedance behavior, including constant-phase-element-like responses, diffusion-related effects, and frequency-dependent conductivity or permittivity. Extending the method to such samples will require broader equivalent-circuit models and additional validation.

With pulse excitation, it is possible to fit the DFT spectrum in the frequency domain, as well as directly fit the time-domain impulse response. The frequency-domain approach is sensitive to spectral leakage, windowing, and noise distortion introduced by the DFT, but the spectral-fitting problem is easier [41,42,43,44]. Time-domain fitting can be problematic for complex systems, but we show that it can be effective for estimating lumped parameters in a simple system.

A neural-network-based estimator trained on model-generated transient responses is used to extract lumped circuit parameters from measured data. Noise and temporal variability are incorporated during training so that the estimator can account for typical waveform variations while providing low-latency parameter extraction. The method is experimentally validated using a configurable lumped-element equivalent circuit and aqueous NaCl solutions of controlled conductivity, with results showing agreement within a few percent of reference values. Owing to its speed, simplicity, and non-galvanic nature, the technique represents a promising low-latency measurement strategy for simplified capacitively coupled impedance sensing, with potential relevance to future CCEIT systems where rapid acquisition of boundary measurements is required.

## 2. Theoretical Model of the Measurement Setup

### 2.1. Equivalent Circuit and Excitation Signal

**Figure 1:**
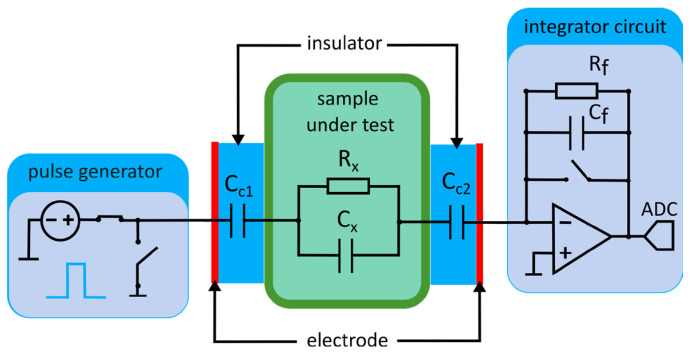
Equivalent circuit of the proposed measurement setup with capacitively coupled electrodes ($C_{c1}$, $C_{c2}$), sample impedance ($R_{x}∥C_{x}$), and integrator front-end.

The circuit used in the proposed impulse-based capacitive impedance measurement method is shown in Figure 1. The sample under test is modeled as a parallel combination of resistance $R_{x}$ and capacitance $C_{x}$, representing its conductive and dielectric properties, respectively. The surface electrodes are not in galvanic contact with the sample; instead, they are capacitively coupled through two interface capacitances, $C_{c1}$ and $C_{c2}$, determined by the electrode geometry, dielectric layer thickness, and the permittivity of the insulating medium.

In the present implementation, these coupling capacitances are formed by dielectric layers placed between the metallic electrodes and the tested medium. However, from the circuit-model perspective, the method does not strictly require the capacitance to originate from a physical dielectric coating on the electrode surface. An equivalent series capacitance could also be introduced electrically in the measurement path, for example by using a discrete capacitor. However, this circuit-equivalent implementation should be distinguished from the contactless capacitive-coupling configuration considered in this work. If the metallic electrode is in direct galvanic contact with the tested medium and the series capacitance is introduced only by an external discrete capacitor, the system should be regarded as a galvanic-contact configuration with an added series capacitance, rather than as a contactless probe. In such a configuration, electrode polarization, interfacial electrochemical reactions, corrosion, and other metal–electrolyte effects may become relevant.

A single rectangular voltage pulse $v_{in}(t)$ with amplitude $V_{0}$ and duration T, produced by the pulse generator shown in Figure 1, is used to excite the system. Such a pulse provides broadband excitation; however, its spectral content must be interpreted with care. For an ideal rectangular pulse of duration T, the Fourier transform has a sinc-shaped spectrum, with the first zero occurring at $f_{0}\;=\;1/T$. Thus, $f_{0}$ characterizes the first zero in the sinc-shaped spectrum imposed by the finite pulse duration. The pulse width, together with the acquisition window, determines which transient time constants can be observed with sufficient sensitivity.

The finite rise time $t_{r}$ of the pulse limits the frequency content associated with the pulse transition. For a first-order bandwidth-limited transition, the 10–90% rise time is commonly related to the equivalent bandwidth by $f_{H}\;\approx \;0.35/t_{r}$ [45]. In the experiments reported here, pulse durations on the order of several tens of microseconds were used. For example, for T = 20 μs and $t_{r}\;=\;200\;ns$, the first spectral zero occurs at $f_{0}\;=\;50\;kHz$, whereas the rise-time-limited equivalent bandwidth is approximately $f_{H}=1.8\;MHz$. Consequently, the pulse should be understood as a finite-duration broadband excitation with a spectrum shaped by both pulse width and finite rise time, rather than as an excitation with an assumed flat frequency band. In the proposed method, the circuit parameters are extracted from the temporal shape of this transient response under the assumed lumped equivalent model.

The front-end integrator (Figure 1), composed of a feedback resistor $R_{f}$ and capacitor $C_{f}$, converts the transient current into a voltage waveform suitable for digital acquisition by the ADC. The recorded waveform carries information about the sample parameters $R_{x}$, $C_{x}$, and the coupling capacitance $C_{c}$. In contrast to conventional AC or frequency-swept methods, this approach relies on the temporal shape of a single transient response.

### 2.2. Expected Transient Response and Analytical Model

The transient voltage response $U_{o}\;(t)$ at the output of the integrator in the equivalent circuit (Figure 1) can be analytically derived using standard Laplace transform techniques. In the feedback branch, the capacitor $C_{f}$ is connected in parallel with a large resistor $R_{f}$. The role of $R_{f}$ is to provide a DC discharge path and prevent long-term saturation of the integrator. Over the short observation window used in this work, however, the capacitive branch dominates the response. In the implemented circuit, $R_{f}=10\;M\Omega$ and $C_{f}=1234\;pF$, giving a feedback time constant of approximately 12.3 ms. Since the analyzed transient window is on the order of tens of microseconds, the leakage through $R_{f}$ has a negligible influence on the short-time waveform. Therefore, the analytical derivation uses the ideal capacitive-integrator approximation. Assuming a single effective coupling capacitance $C_{c}$ representing the series combination of $C_{c1}$ and $C_{c2}$, and modeling the sample as a parallel network $R_{x}∥C_{x}$, the output voltage response to a rectangular excitation pulse can be expressed as follows [46]:

(1) $$U_{o}\left(t\right)=\;\frac{U_{i}}{C_{f}}\left[\frac{C_{c}C_{x}}{C_{c}+C_{x}}+\frac{{C_{c}}^{2}}{C_{c}+C_{x}}\left(1-e^{\frac{-t}{\tau }}\right)\right].$$

In the present lumped model, $C_{c}$ is interpreted as an effective parameter of the complete measurement setup, including the dominant contribution of the dielectric insulation between the electrode and the sample, as well as unavoidable parasitic contributions from the electrode structure, cabling, and input stage. These additional contributions are not represented as separate circuit elements in the simplified analytical model. The effective time constant of the system is given by:

(2) $${{\tau =R}_{x}(C}_{c}+C_{x}).$$

The first term in brackets in (1) represents the instantaneous capacitive response determined by $C_{c}$ and $C_{x}$, while the exponential term accounts for the gradual charging of the resistive–capacitive branch through $R_{x}$.

The analytical expression also assumes an ideal capacitive integrator and ideal switching of the excitation pulse. In the physical circuit, the measured transient may additionally be affected by the finite gain-bandwidth product and input capacitance of the operational amplifier, switch on-resistance, switch off-capacitance, leakage paths, PCB and cable parasitics, and the finite bandwidth of the acquisition chain. Some capacitance-like parasitic contributions may be absorbed into the effective coupling capacitance $C_{c}$, whereas other nonidealities affect the waveform in a way that is not strictly equivalent to a single series capacitance. In the present work, these effects are not introduced as separate parameters in the analytical model; instead, the model describes the dominant transient behavior using a compact set of effective circuit parameters.

**Figure 2:**
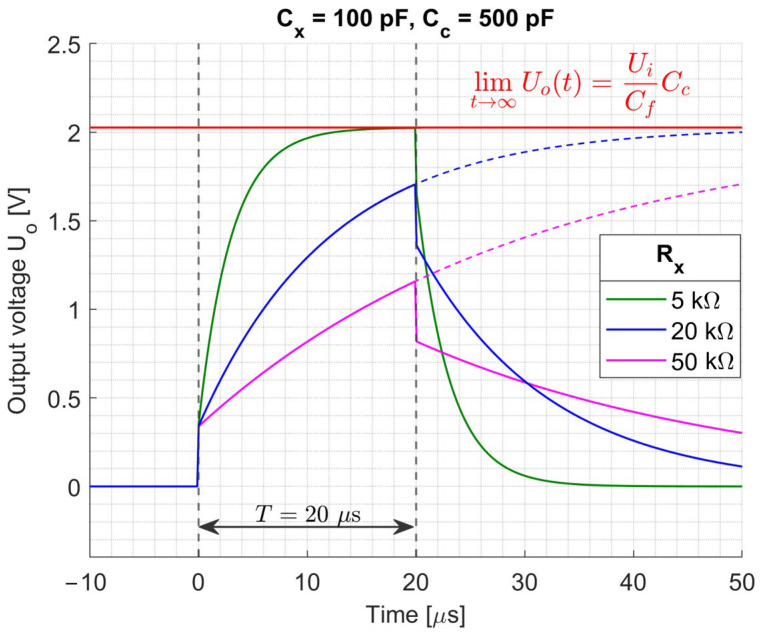
Analytical transient waveforms of the output voltage $U_{o}\;(t)$ predicted by the equivalent-circuit model for three values of $R_{x}$, while $C_{x}=100\;pF$ and $C_{c}=500\;pF$ were kept constant. The model parameters were $U_{i}=5\;V$, $C_{f}=1234\;pF$, and pulse width T=20 μs.

The transient waveforms presented in Figure 2 were calculated from the analytical expression in (1) for three different values of $R_{x}$, while $C_{x}$ and $C_{c}$ were kept constant. These model-predicted waveforms illustrate the influence of the sample resistance on the transient response. The rising edge of each waveform consists of two distinct regions. The leading edge corresponds to the fast capacitive coupling effect, primarily governed by the combined capacitance. The slower rise region, or crest phase, follows with a characteristic time constant τ, reflecting the resistive charging dynamics through $R_{x}$. After the excitation pulse ends, the signal decays as the stored charge in $C_{x}$ is discharged through the same path. The shape of the transient waveform is thus influenced by all three parameters: $C_{x}$ determines the overall curvature of the response, $C_{c}$ affects its initial amplitude, and $R_{x}$ controls the slope and duration of the slower exponential rise.

The analytical expression in (1) also allows evaluating the steady-state behavior of the circuit. In the limit of an infinitely long excitation pulse, the output voltage saturates to a value that depends solely on the coupling capacitance:

(3) $$\underset{t\to \infty }{\lim}U_{o}\left(t\right)=C_{c}\frac{U_{i}}{C_{f}}.$$

This means that if the excitation were applied indefinitely, the signal would contain no information about the sample impedance ($R_{x}$, $C_{x}$), but only about the isolation capacitance $C_{c}$. Therefore, all useful information about the sample is encoded in the transient part of the response preceding saturation.

To enable practical parameter extraction, the measured waveform can be approximated by a general exponential function of the form:

(4) $$y\left(t\right)=\;\alpha +\beta \left(1-e^{\frac{-t}{\tau }}\right).$$

where α, β, and the already defined time constant τ are coefficients describing the transient waveform. These coefficients can be obtained by fitting this empirical form to the transient response, for example using the least-squares method. By comparing this empirical representation with the analytical solution of the equivalent-circuit model, the following relationships between the waveform coefficients and the circuit parameters can be established:

(5) $$\alpha =\frac{U_{i}}{C_{f}}\frac{C_{c}C_{x}}{C_{c}+C_{x}},$$

(6) $$\beta =\frac{U_{i}}{C_{f}}\frac{{C_{c}}^{2}}{C_{c}+C_{x}}.$$

Based on these parameters, the coupling capacitance can be derived as:

(7) $$C_{c}=\frac{C_{f}}{U_{i}}\left(\alpha +\beta \right),$$

and subsequently, the impedance components of the medium under test can be obtained as:

(8) $$R_{x}=\frac{\tau }{C_{c}\left(1+\frac{\alpha }{\beta }\right)},$$

(9) $$C_{x}=\frac{\alpha }{\beta }C_{c}.$$

These relationships show that, within the ideal equivalent-circuit model, the transient waveform contains information about all three equivalent-circuit parameters. In the parameter-estimation procedure used in this work, $R_{x}$, $C_{x}$, and $C_{c}$ are treated as jointly estimated unknowns, and the complete waveform is used for parameter extraction. This joint estimation is performed using the neural-network estimator and, in the benchmark analysis, by nonlinear least-squares fitting of the analytical model. Thus, the proposed approach uses a single-pulse time-domain response, without requiring a frequency sweep.

After the excitation pulse is switched off at t=T, the input voltage returns to zero and the stored charge in $C_{c}$ and $C_{x}$ discharges through the sample resistance $R_{x}$. The output voltage then decays exponentially according to the following formula:

(10) $$U_{o}\left(t\right)=\frac{U_{i}}{C_{f}}\frac{{C_{c}}^{2}}{C_{c}+C_{x}}\left(1-e^{\frac{-T}{\tau }}\right)\;e^{\frac{-t-T}{\tau }},\;t>T,$$

with the time constant τ defined in (2).

This discharge phase can also be used for parameter estimation, as the decay constant provides an independent measure of the resistive component $R_{x}$. In practical implementations, both the charging and discharging parts of the transient may be analyzed jointly to improve accuracy and noise robustness.

### 2.3. Influence of the Coupling Capacitance

The coupling capacitance $C_{c}$ is a key parameter governing both the amplitude and the temporal dynamics of the measured transient. As expressed in (2), it directly affects the system time constant τ. An increase in $C_{c}$ slows down the transient response and increases its steady-state amplitude, whereas a small $C_{c}$ results in a fast, sharply peaked waveform dominated by the capacitive term.

If $C_{c}$ is too small, the signal saturates rapidly, and most of the recorded samples correspond to the flat region of the curve, where sensitivity to $R_{x}$ is minimal. Conversely, if $C_{c}$ is too large, the transient becomes nearly linear during the excitation window T, and the exponential rise characteristic is not fully developed, reducing the observability of $R_{x}$ and $C_{x}$.

The favorable conditions occur when the exponential growth is substantially, but not entirely, completed within the excitation period. Assuming a first-order transient of the form $1-e^{-t/\tau }$, the signal reaches approximately 95% of its steady-state value at t=3τ. Therefore, for a rectangular pulse of duration T, the most informative waveform is obtained when:

(11) $$\tau \approx \frac{T}{3}.$$

This relation ensures that the measured signal covers both the initial capacitive phase and the main exponential region without entering the steady-state saturation zone. An optimal coupling capacitance yields a well-defined exponential curve that can be accurately sampled and fitted.

In practice, $C_{c}$ also determines the usable dynamic range of the integrator output; excessively large $C_{c}$ may cause saturation at the amplifier or ADC input, although this is a secondary limitation compared with the information content of the transient itself.

### 2.4. Parameter Estimation Method

Conventional nonlinear least-squares fitting can provide accurate parameter estimates when the assumed equivalent circuit is appropriate and the optimizer is properly initialized. However, repeated iterative fitting may become less convenient for high-throughput or embedded measurements, particularly when robustness to noise, waveform distortions, and timing uncertainty is required.

In the proposed system, each measurement is performed within a few tens of microseconds. Therefore, a parameter-estimation method with very low inference latency is desirable, especially for future real-time or multi-channel implementations. For this reason, a feedforward neural network was trained to learn the nonlinear mapping between transient voltage waveforms and the corresponding circuit parameters. The network is not used as a replacement for the analytical model, but as a fast surrogate inverse mapping trained on transients generated from this model. Once trained, the network performs the estimation through a single forward pass, effectively transferring the computational cost from the measurement stage to the offline training stage. This enables rapid reconstruction of impedance parameters directly from measured transients while avoiding repeated optimization for each individual waveform.

The input vector to the network consists of uniformly sampled voltage transients simulated using the analytical models (1) and (2). Synthetic data were generated across a wide range of physical parameters to ensure generalization: $R_{x}\in [0.1,\;100]\;k\Omega$, $C_{x}\in [1,\;100]\;pF$, $C_{c}\in [500,\;900]\;pF$. The parameters $R_{x}$ and $C_{x}$ were sampled on a logarithmic scale, while $C_{c}$ was drawn linearly within its range. A total of 500,000 transients were simulated, each perturbed with additive Gaussian noise (σ=0.005) to mimic measurement imperfections. This training strategy allows noise-related waveform variability to be incorporated into the estimator during training rather than handled separately during each individual fit. The dataset was split into 80% for training and 20% for validation to monitor convergence and prevent overfitting.

The fitting neural network comprised three fully connected layers with 128, 64, and 32 neurons, each followed by a ReLU activation, and a final linear output layer with three neurons corresponding to ($R_{x}$, $C_{c}$, $C_{x}$). Training was performed in MATLAB R2025b (Deep Learning Toolbox, The MathWorks, Inc., Natick, MA, USA) using the Adam optimizer with learning rate $10^{-3}$, mini-batch size of 64, and a maximum of 40 epochs. GPU acceleration was used to shorten training time.

Once trained, the network estimates the three circuit parameters directly from a single measured transient through one forward pass. This approach enables low-latency parameter extraction and is suitable for repeated or future multi-channel measurements, while the inclusion of noise and temporal variability during training exposes the estimator to typical waveform perturbations.

For comparison with a conventional fitting-based approach, the same transient model was also fitted using nonlinear least-squares fitting with the Levenberg–Marquardt algorithm (NLLS). The fitted parameters were $R_{x}$, $C_{x}$, and $C_{c}$. The optimization was performed in the log10-transformed parameter space to account for the different orders of magnitude of the parameters and to preserve their positivity. The lower and upper bounds were the same as the parameter ranges used for neural-network training. The initial parameter vector was selected as the geometric center of these ranges. The residuals were calculated between the measured transient segment and the model-predicted waveform and normalized by the signal amplitude. The NLLS procedure was used as a conventional fitting benchmark, whereas the neural network was retained as the primary estimator for low-latency repeated measurements.

## 3. Configurable Lumped-Element Equivalent Circuit for Model Verification

**Figure 3:**
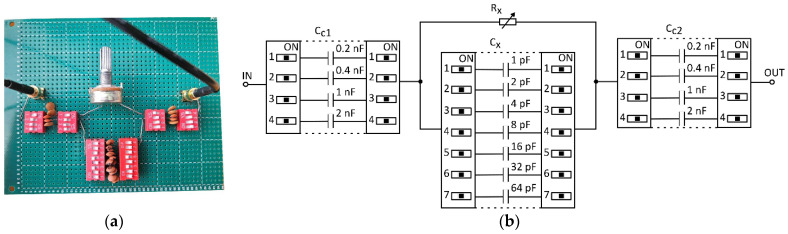
Configurable lumped-element equivalent circuit used for model verification: (**a**) photograph of the assembled circuit, (**b**) schematic diagram showing the configurable elements $R_{x}$, $C_{x}$, and $C_{c}$ and the switching matrix used to select different impedance configurations.

To verify the analytical model described in Section 2, a configurable lumped-element equivalent circuit (Figure 3) was constructed to reproduce the behavior of the measured sample under controlled conditions. The phantom allows independent adjustment of the sample resistance $R_{x}$, internal capacitance $C_{x}$, and coupling capacitance $C_{c}$, thus providing a versatile platform for validating theoretical relationships.

The resistive component $R_{x}$ is simulated by a precision potentiometer, while the capacitive elements $C_{x}$ and $C_{c}$ are implemented using high-stability film capacitors. Two capacitors of equal value (1% tolerance) connected in series represent the coupling interfaces $C_{c1}$ and $C_{c2}$. Each capacitor and resistor can be selectively included or bypassed using electronic switches, allowing multiple impedance configurations without rewiring. This arrangement makes it possible to reproduce different combinations of $R_{x}$, $C_{x}$, and $C_{c}$ corresponding to both conductive and predominantly capacitive samples. In the implemented prototype, $R_{x}$ can be set between 10 kΩ and 90 kΩ, the internal capacitance $C_{x}$ is programmable from 1 pF to 127 pF, and each coupling capacitor $C_{c1}$ and $C_{c2}$ can be selected to 0.2 nF, 0.4 nF, 1 nF, or 2 nF, defining the effective coupling capacitance $C_{c}$. The reference values of $R_{x}$, $C_{x}$, and $C_{c}$ used for validation were determined independently using an Agilent 4263B LCR meter (Agilent Technologies, Santa Clara, CA, USA) at 10 kHz before the transient measurements. These values served as reference parameters for the lumped equivalent-circuit validation. In this validation experiment, the purpose of the LCR-meter measurement was to independently determine the values of the discrete circuit elements in the controlled setup. The transient method then estimated the corresponding effective lumped parameters from the finite-duration time response. Thus, the comparison with LCR-meter measurements validates the consistency of the estimated parameters with independently measured circuit-element values.

The experimental setup consists of a pulse generator and the integrator circuit, as shown in Figure 1. The circuit was driven by rectangular voltage pulses of amplitude $v_{in}=5\;V$ and duration T=20 μs. The output voltage was measured using the integrator front-end described by the analytical model, with feedback components $R_{f}$ and $C_{f}$ selected to ensure linear operation over the expected dynamic range. The feedback resistor $R_{f}=10\;M\Omega$ provided a DC discharge path and prevented long-term saturation of the integrator. Together with $C_{f}$ it formed a feedback time constant of approximately 12.3 ms, which is much longer than the analyzed transient window. Therefore, its influence of $R_{f}$ on the short-time waveform was neglected in the analytical model. The resulting transients were digitized at a sampling rate of 10 MHz for subsequent comparison with simulated signals.

A series of measurements was carried out using the configurable equivalent circuit described above. For each analyzed configuration of the equivalent circuit, 500 repeated transient measurements were acquired and used to assess repeatability and estimate the standard deviation.

**Figure 4:**
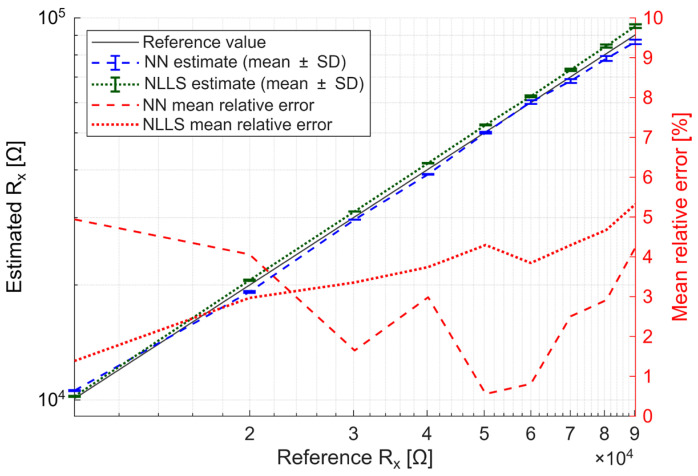
Estimated resistance $R_{x}$ compared with the reference values for $C_{x}=60\;pF$ and $C_{c}=500\;pF$. The blue and green markers show the NN and NLLS estimates, respectively. The thin gray line indicates the reference value, while the red dashed and dotted lines show the corresponding mean relative errors.

**Figure 5:**
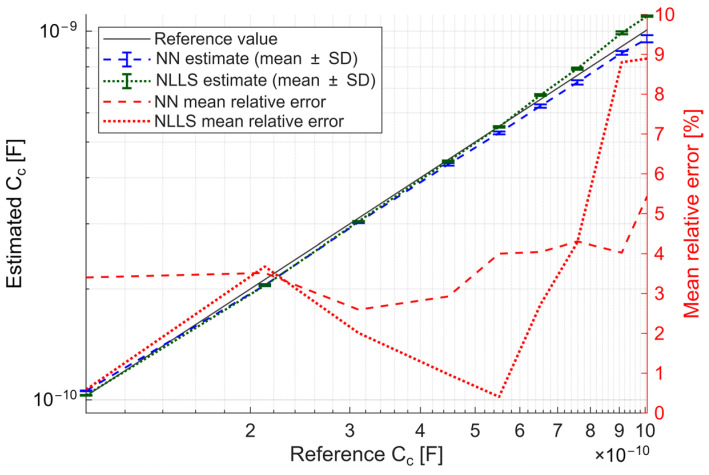
Estimated coupling capacitance $C_{c}$ compared with the reference values for $R_{x}=50\;k\Omega$ and $C_{x}=60\;pF$. The blue and green markers show the NN and NLLS estimates, respectively. The thin gray line indicates the reference value, while the red dashed and dotted lines show the corresponding mean relative errors.

**Figure 6:**
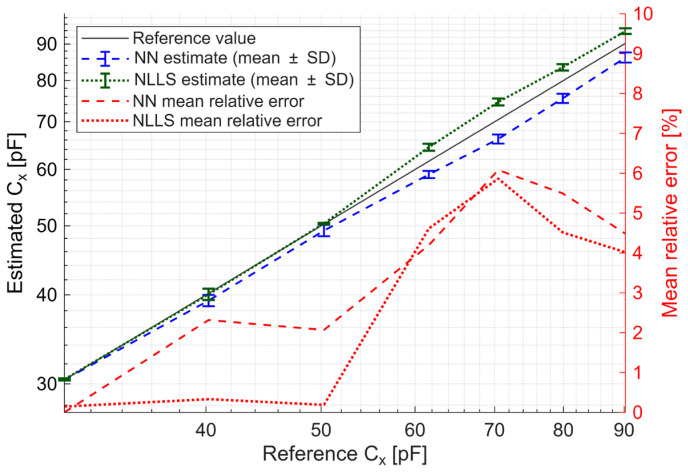
Estimated sample capacitance $C_{x}$ compared with the reference values for $R_{x}=50\;k\Omega$ and $C_{c}=500\;pF$. The blue and green markers show the NN and NLLS estimates, respectively. The thin gray line indicates the reference value, while the red dashed and dotted lines show the corresponding mean relative errors.

Figure 4, Figure 5 and Figure 6 compare the parameters estimated from the measured transients with the reference values determined independently using the Agilent 4263B LCR meter. The same recorded transients were processed using both the neural-network estimator and nonlinear least-squares fitting (NLLS), used here as a conventional fitting benchmark. In each figure, the left axis shows the estimated parameter value, whereas the right axis shows the corresponding mean relative error. The thin gray line represents the reference value. The blue and green markers denote the NN and NLLS estimates, respectively, calculated as mean values from repeated transient measurements; vertical bars indicate one standard deviation. These standard deviations should be interpreted as empirical estimates of the repeatability of the complete measurement-and-estimation procedure, rather than as model-derived confidence intervals for individual parameter estimates.

As shown in Figure 4, Figure 5 and Figure 6, both estimators follow the reference values over the tested ranges. For the resistance measurement, the relative errors remain within a few percent for both approaches. Similar behavior is observed for the coupling capacitance measurement, although the error increases at the highest $C_{c}$ values. In the sample-capacitance case, the NN and NLLS estimates again show errors of the same order, with the largest deviations appearing for higher $C_{x}$ values. These results confirm that the analytical model can be fitted directly using NLLS and that the neural-network estimator provides comparable reconstruction accuracy within the tested range.

**Figure 7:**
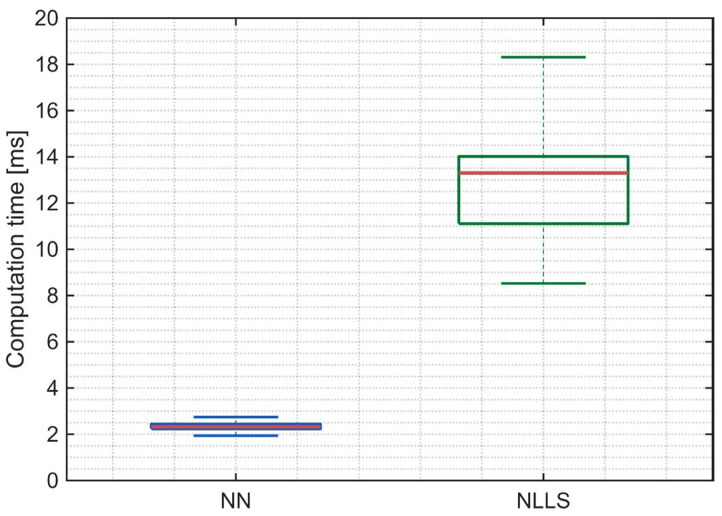
Computation-time comparison between the neural-network estimator and nonlinear least-squares fitting (NLLS). Boxplots show the distribution of processing times for the transient waveforms acquired from the configurable lumped-element equivalent circuit. Both estimators were evaluated in MATLAB on the same workstation using CPU execution.

The computation time comparison in Figure 7 shows the practical motivation for retaining the neural-network estimator as the primary parameter-extraction method. Both estimators were evaluated under the same MATLAB CPU-based implementation conditions and using the same set of transient waveforms. The NLLS procedure required a longer and more variable processing time, which is consistent with its iterative character and with the waveform-dependent number of Levenberg–Marquardt iterations required for convergence. In contrast, the neural-network estimator performs a single forward pass, resulting in a shorter and more consistent processing time. Therefore, the neural network was retained not because direct NLLS fitting is impossible, but because it provides deterministic low-latency inference for repeated measurements and future multi-channel implementations.

**Figure 8:**
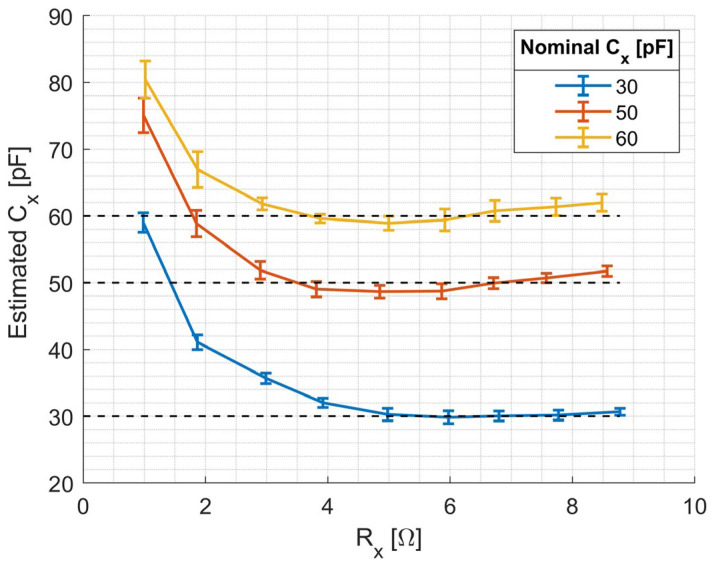
Effect of $R_{x}$ on the deviation of the estimated capacitance $C_{x}$ under $C_{c}\;=\;500\;pF$ and different nominal values of $C_{x}$. The dashed horizontal lines indicate the nominal capacitance levels, while the plotted points with error bars show the estimated mean values and their variability as a function of $R_{x}$.

The influence of $R_{x}$ on the accuracy of $C_{x}$ estimation is shown in Figure 8. In this experiment, $C_{c}$ was kept constant at 500 pF, while $R_{x}$ was varied to evaluate how the transient time constant affects the observability of $C_{x}$. For low $R_{x}$ values, the time constant defined in (2) becomes short, so the resistance-related exponential part of the transient starts to rise rapidly immediately after the initial capacitive step. As a result, the boundary between the fast capacitive component and the early part of the resistance-related exponential rise becomes less distinct. In this regime, the estimator may attribute part of the rapid early exponential increase to a larger capacitive contribution, which leads to an overestimation of $C_{x}$. This interpretation is consistent with the systematic positive deviation of the estimated $C_{x}$ observed for low $R_{x}$ values in Figure 8.

In addition, as follows from (2), the influence of $C_{x}$ on the time constant is reduced when $C_{c}$ is larger than $C_{x}$. Thus, $C_{x}$ has only a limited independent effect on the slower part of the waveform, and most of the information related to $C_{x}$ is concentrated in the fast leading region of the transient. This region is particularly sensitive to finite excitation rise time, analog bandwidth, sampling resolution, and timing uncertainty. Therefore, shorter excitation rise time, wider analog bandwidth, and faster or non-uniform sampling may improve the observability of $C_{x}$. However, these improvements cannot fully eliminate the coupling between $R_{x}$, $C_{x}$, and $C_{c}$, which leads to systematic deviations in the estimated $C_{x}$. In the tested configuration with $C_{c}\;=\;500\;pF$, $C_{x}$ estimation became quantitatively acceptable for $R_{x}$ values above approximately 40 kΩ; in this range, the largest observed deviation was about 2 pF, corresponding to a relative error of approximately 6.7% for the $C_{x}\;=\;30\;pF$ setting.

This threshold is specific to the selected coupling capacitance and the adopted measurement configuration. Increasing $C_{c}$ generally increases the capacitive contribution to the measured transient and extends the effective time constant, which can improve the recovery of $C_{x}$ for lower $R_{x}$ values. However, the effect is not necessarily beneficial over the entire parameter range. For high $R_{x}$ values, an excessively large $C_{c}$ may overextend the transient, increase parameter coupling, increase the output amplitude, or bring the system closer to the dynamic limits of the analog front-end.

The coupling capacitance is determined by the electrode geometry, dielectric thickness, dielectric permittivity, and electrode–sample positioning. In a simplified parallel-plate approximation, $C_{c}$ scales approximately inversely with the effective separation distance. Therefore, increasing the dielectric thickness or electrode–sample distance decreases $C_{c}$, reduces the capacitive contribution to the transient response, and may narrow the range in which $C_{x}$ can be reliably estimated. However, the real probe geometry also includes fringing fields, finite electrode dimensions, and parasitic capacitances, so the parallel-plate expression should be treated as a first-order design estimate. This is consistent with the interpretation of $C_{c}$ as an effective coupling parameter of the complete measurement setup.

This introduces a practical design trade-off. A larger dielectric thickness or electrode–sample separation improves physical isolation and preserves the contactless character of the probe but reduces the coupling capacitance. Conversely, reducing the dielectric thickness increases $C_{c}$ and improves capacitive coupling, but may increase sensitivity to mechanical positioning, parasitic capacitances, and front-end dynamic-range limitations. Therefore, the coupling geometry should be selected with respect to the expected $R_{x}$ and $C_{x}$ ranges, required isolation, pulse duration, sampling rate, and analog front-end limits.

Overall, the results confirm that the proposed method and numerical model can accurately determine the impedance parameters within the tested range, while maintaining low measurement dispersion across repeated trials.

## 4. Experimental Validation on Saline Solutions

**Figure 9:**
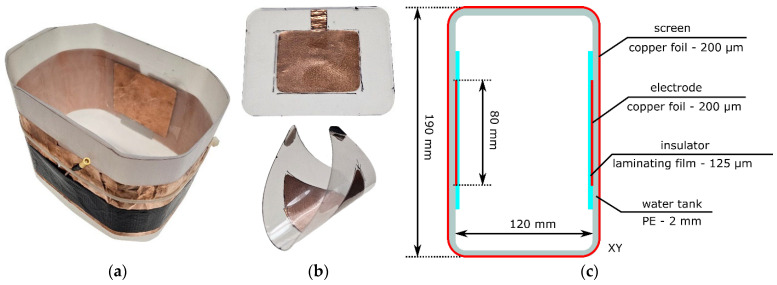
Measurement probe used for saline-solution experiments: (**a**) photo of the sensor; (**b**) elastic electrode after lamination; (**c**) schematic diagram.

To verify the practical applicability of the proposed method, a measurement probe was specifically designed and fabricated for this experiment. The probe consisted of a waterproof container equipped with surface electrodes adhered to its inner walls. A photograph and a cross-sectional schematic of the probe are shown in Figure 9. The copper-foil electrodes were electrically insulated using a laminating process. The laminating film, composed of ethylene–vinyl acetate (EVA) and polyethylene terephthalate (PET), has a relative permittivity ranging from 2.5 to 3. The thickness of the insulation layer was 125 µm for the first probe and 125 µm for the second. The electrodes, each measuring 80 mm × 80 mm, were positioned on opposite container walls, separated by 120 mm. Each container had an internal width of 190 mm and a total volume of approximately 3.5 L.

The experimental procedure involved filling the measurement container with 3.5 L of demineralized water. Subsequently, small portions of a 0.9% NaCl physiological saline solution (Symphar Sp. z o.o., Warsaw, Poland) were incrementally added using a precision pipette to gradually increase the conductivity of the medium over the investigated range. Before the experiments, the distilled water and saline solution were stored for an extended time in the same laboratory environment to ensure thermal equilibration. During the saline-solution measurements, the sample temperature was monitored using the temperature sensor integrated with the reference conductivity measurement setup and remained stable at room temperature. Therefore, the reference conductivity values and the transient measurements were obtained under the same stable temperature conditions. After each addition, 500 repeated transient measurements were acquired for the given saline concentration and used to calculate the mean value and standard deviation of the estimated parameters.

**Figure 10:**
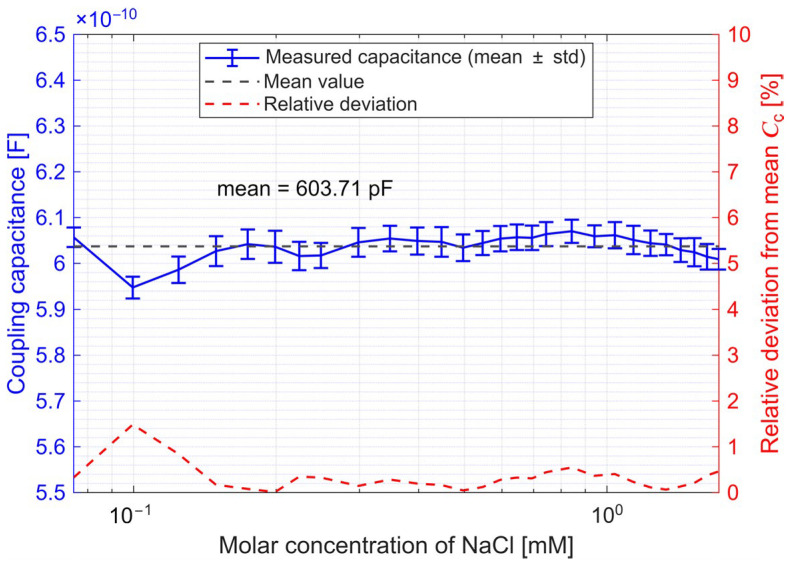
Coupling capacitance $C_{c}$ as a function of NaCl molar concentration during incremental saline dilution. The blue markers represent the mean values ± standard deviation obtained from repeated measurements. The dashed black line indicates the overall mean value ($\bar{C_{c}}\approx 601\;pF$), while the red curve (right axis) shows the relative systematic deviation from this mean.

Figure 10 presents the evolution of the extracted contact capacitance $C_{c}$ as a function of the molar concentration of NaCl. Since the probe geometry, electrode area, laminate thickness, and immersion state were kept constant during the concentration sweep, the effective coupling capacitance $C_{c}$ was expected to remain approximately constant. The near-constant values observed in Figure 10 are consistent with this assumption. Small deviations in the extracted $C_{c}$ values should therefore not be interpreted solely as physical changes in the coupling interface. They may also reflect temperature changes, incomplete wetting, air bubbles, slight changes in the effective dielectric environment, or variations in estimation accuracy across different $R_{x}$ ranges.

**Figure 11:**
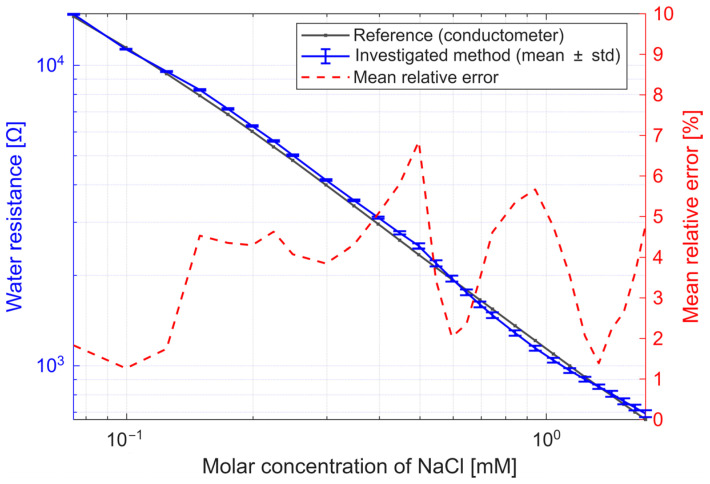
Estimated sample resistance $R_{x}$ as a function of NaCl molar concentration compared with reference conductometer readings. The blue markers represent the mean values ± standard deviation obtained from repeated measurements, while the solid black line shows the reference data. The red dashed curve (right axis) presents the approximation error of the proposed method.

Figure 11 shows the estimated sample resistance $R_{x}$ and the corresponding approximation error with respect to the reference conductometer measurements, both plotted as functions of NaCl molar concentration. The left axis represents the resistance values, while the right axis indicates the approximation error.

Reference measurements were carried out using a CC-404 Conductometer (Elmetron, Zabrze, Poland), offering an accuracy of up to 1 µS/cm. To validate the experimental results, a numerical model of the probe was prepared using our in-house MATLAB toolbox, ECTsim [47]. This model was used to calculate the expected resistance values corresponding to the specific conductivities measured with the conductometer during the salting process.

The results indicate that the contact capacitance $C_{c}$ remains nearly constant across the entire concentration range (mean value ≈ 601 pF), confirming the stability of the coupling interface. This value is also in good agreement with the theoretical estimation based on the geometry of the electrodes and the dielectric layer. Assuming a parallel-plate model with two identical insulating films (each of thickness d=125 μm) covering electrodes of 80 mm×80 mm area, and a relative permittivity of polyethylene ($\varepsilon_{r}\approx 2.64$), the equivalent series capacitance is given by:

(12) $$C_{c}=\frac{\varepsilon_{0}\varepsilon_{r}A}{2d}=\frac{8.854\times 10^{-12}\times 2.3\times 6.4\times 10^{-3}}{2\times 125\times 10^{-6}}\;\approx 601\;pF,$$

which closely matches the experimentally obtained value.

In contrast, the measured resistance $R_{x}$ decreases exponentially with increasing NaCl concentration, following the trend obtained from the reference conductometer. The proposed method was experimentally validated for solution resistances ranging from approximately 14.8 kΩ to 0.68 kΩ, corresponding to NaCl molar concentrations between 0.075 mM and 1.72 mM. Within this range, the relative error did not exceed about 7%, and the standard deviation of repeated measurements was typically around 32 Ω, confirming that the simplified two-electrode capacitive configuration provides stable and repeatable estimation of solution conductivity.

Although the equivalent model includes both the resistive $R_{x}$ and capacitive $C_{x}$ components of the tested medium, a reliable estimation of $C_{x}$ was not achieved for the conductivity range investigated in the saline-solution experiments. This represents an important limitation of the present implementation. In electrolyte measurements, capacitive effects may arise from several mechanisms, including bulk dielectric response, interfacial polarization, and electrode-related phenomena. In the tested configuration, the coupling capacitance $C_{c}$ provided by the laminated insulation was relatively small, and the capacitive contribution of the sample occupied only a minor part of the total transient response compared with the subsequent exponential rise governed mainly by $R_{x}$. As a result, the fitting or neural-network-based estimation became dominated by the resistive component, while the extracted $C_{x}$ values were strongly affected by noise and parameter coupling. This behavior was particularly evident for $R_{x}$ values below approximately 14 kΩ, corresponding to the saline-solution measurements. Consequently, the present experimental validation should be interpreted primarily as a demonstration of contactless conductivity estimation, whereas reliable extraction of $C_{x}$ will require either larger coupling capacitance, shorter and better-resolved transients, or an optimized front-end and excitation scheme.

## 5. Discussion

The proposed time-domain impedance measurement method was experimentally verified for solutions with conductivities up to approximately 0.03 S/m, corresponding to a minimum measured resistance of about 680 Ω in the applied geometry. No integrator saturation was observed within this tested range, and the resistance-related estimates remained within the reported error range. However, the absolute upper conductivity limit of the current sensor configuration was not determined experimentally, because the measurements were not continued until integrator saturation or loss of reliable $R_{x}$ extraction occurred. The upper conductivity limit is not a universal property of the method alone, but depends on the excitation amplitude and rise time, analog bandwidth, feedback capacitance, coupling capacitance, integrator output range, and the required estimation accuracy.

This range covers the conductivity of weak saline solutions but remains lower than that of typical biological tissues or physiological fluids (∼1 S/m). Moreover, the present validation was performed using a simplified three-parameter lumped equivalent-circuit model, which captures the effective sample resistance, sample capacitance, and electrode coupling capacitance. Real biological tissues and physiological fluids may exhibit dispersive and non-ideal impedance behavior, including constant-phase-element-like responses, diffusion-related effects, and frequency-dependent conductivity or permittivity. These effects are not explicitly represented in the current model.

Under the validated operating conditions, the present implementation can be interpreted as a reliable estimator of resistance-related parameters and coupling capacitance, whereas the recovery of $C_{x}$ remains conditional on sufficiently well-resolved transients and requires further validation for conductive saline media. At higher conductivities, the transient response becomes faster and the capacitive contribution becomes more difficult to separate from the dominant resistive component, especially with the present 10 MHz sampling rate and finite excitation rise time. This increases parameter coupling and limits the reliable extraction of $C_{x}$, particularly in highly conductive media.

Considering these limitations, a realistic first imaging-oriented application of the proposed approach may be conductivity-related CCEIT, where rapid acquisition of boundary measurements is required for image reconstruction. In this case, the reconstructed contrast would initially correspond mainly to effective conductivity changes, rather than to full complex-impedance information. Reliable recovery of both conductivity- and permittivity-related components over a broad range of sample conductivities will require further development of the excitation strategy, front-end electronics, sampling rate, and equivalent-circuit model.

In the case of higher conductivity, which is expected in biomedical or physiological measurements, the time constant τ decreases, making the measurement process more challenging. Two potential strategies can be employed to enable accurate sensing under such conditions.

The first approach is to increase the contact capacitance $C_{c}$. However, this is difficult in practice because materials with higher relative permittivity $\varepsilon_{r}$ (such as ceramics or composites) usually lack the required mechanical flexibility. Conversely, producing polymer-based insulating layers (films or coatings) that are sufficiently thin (on the order of a few micrometers) while maintaining adequate resistance to wear and fluid absorption remains technologically demanding. Although both approaches are feasible, they are considerably more complex to implement than simple lamination films. A discrete capacitor inserted in series with the measurement path can also be used to control the effective coupling capacitance, especially in laboratory or phantom experiments. However, if this implementation requires direct contact between a metallic electrode and the tested medium, the system no longer remains fully contactless, and electrode polarization, interfacial chemical reactions, and corrosion may become relevant.

The second approach is to increase the sampling rate, potentially beyond 10 MHz if required by the expected transient duration. A higher sampling frequency allows more samples to be acquired along the signal’s rising edge and may improve the observability of fast transients. It may also allow the excitation pulse to be shortened, reducing the number of samples transferred per measurement, particularly in multi-channel systems such as impedance tomographs. However, this strategy requires careful high-speed design of the complete measurement chain. At such frequencies, the analog front-end bandwidth, PCB layout, parasitic effects, cable impedance, impedance matching, and possible signal reflections must be taken into account. Consequently, higher sampling rates may improve parameter recovery but at the cost of increased hardware complexity and system expense.

## 6. Conclusions

This work has introduced a simplified complementary method for capacitive impedance measurement based on a single-pulse rectangular excitation and capacitively coupled electrodes. In contrast to techniques with sinusoidal excitation, the proposed approach derives resistive and capacitive parameters from the transient response to an impulse. A key feature of the method is the explicit analytical modeling of the coupling capacitance and estimation of its value, together with the sample parameters. To enable low-latency parameter extraction, a neural-network-based estimator trained on model-generated transient responses was used as a fast surrogate inverse mapping from measured waveforms to lumped circuit parameters.

The method was validated using both a configurable equivalent impedance circuit and NaCl solutions of controlled conductivity. In the reported experiments, resistance-related parameters were estimated with errors on the order of 2–8%, while the coupling capacitance in the saline probe remained stable around 601 pF. The results also define the current operating limitations of the method. Reliable recovery of $C_{x}$ was possible only in a sufficiently high-resistance regime of the tested sample. Under the present settings, for $C_{c}\;=\;500\;pF$, reliable estimation of $C_{x}$ was obtained only for $R_{x}$ values above approximately 40 kΩ.

These findings indicate the present implementation is most directly applicable to rapid resistance- or conductivity-related parameter estimation and coupling-capacitance monitoring within the validated operating range. This makes the method potentially relevant to future capacitively coupled electrical impedance tomography (CCEIT) systems, where rapid acquisition of boundary measurements is required for image reconstruction. However, the method should be regarded as complementary to full EIS rather than as its replacement when detailed frequency-resolved spectral information or low-resistance/high-capacitance regimes must be resolved.

Further work will focus on extending the model and hardware toward full complex-impedance characterization, higher conductivities, dispersive tissue behavior, and broader biological validation. More realistic bioimpedance models may require frequency-dependent elements, such as constant phase elements, diffusion-related impedance terms, or multi-time-constant RC branches, together with additional identifiability analysis and experimental validation. Reaching physiologically relevant conductivities, on the order of 1 S/m, will also require substantial hardware optimization, including shorter excitation rise times, higher analog bandwidth, faster or non-uniform sampling strategies, and optimized dielectric geometry and coupling capacitance. Non-uniform or adaptive sampling may be particularly useful for concentrating temporal resolution in the initial part of the transient, where the capacitive contribution is most pronounced, without requiring uniformly high sampling rates over the entire acquisition window. These developments are necessary because the transient response becomes faster and the capacitive contribution becomes less observable at higher conductivities.

## Data Availability

The raw data supporting the conclusions of this article will be made available by the authors upon request.
